# Filaggrin failure – from ichthyosis vulgaris to atopic eczema and beyond

**DOI:** 10.1111/bjd.14997

**Published:** 2016-09-26

**Authors:** W.H.I. McLean

**Affiliations:** ^1^ Division of Biological Chemistry and Drug DiscoveryCentre for Dermatology and Genetic MedicineSchool of Life SciencesUniversity of DundeeDundeeDD1 9SYU.K

## Abstract

The main proteinaceous component of the keratohyalin granules within the granular layer keratinocytes of the epidermis is the giant, repetitive polyprotein profilaggrin. When granular layer cells commit to terminal differentiation to form the flattened squames of the stratum corneum, profilaggrin is rapidly cleaved into multiple copies of the 37 kDa filaggrin monomer, which binds to and condenses the keratin cytoskeleton, thereby facilitating cellular compression. Within the stratum corneum, filaggrin is broken down to form natural moisturising factor, a pool of amino acids and derivatives thereof that exerts multiple effects. Filaggrin is therefore essential for normal stratum corneum biogenesis and physiology. In 2006, the McLean group identified the first loss‐of‐function mutations in the filaggrin gene (*FLG*) as the cause of the common monogenic genodermatosis ichthyosis vulgaris (IV). In parallel, they showed by multiple methods that these mutations, carried by up to 10% of various human populations are the major genetic predisposing factor for atopic dermatitis (eczema) and all of the associated allergic phenotypes that constitute the atopic diathesis. This paradigm‐shifting work showed that skin barrier deficiency is a major early event in the pathophysiology of eczema and allergy.

## Abstract 2

**What is already known?**

- Filaggrin is a critically important, multifunctional protein required for the normal biogenesis and physiology of the stratum corneum.

**What does (recent) work tell us?**

- Loss‐of‐function mutations in the filaggrin gene are common in the human population and cause clinically dry skin, ichthyosis vulgaris and importantly, are a major genetic predisposing factor for atopic dermatitis and its associated allergic conditions. This work revealed that skin barrier deficiency is the major pathomechanism underlying atopic disease.

Ichthyosis vulgaris (IV) – a dry flaky skin condition frequently seen by all dermatologists – is arguably the most common monogenic hereditary skin disorder.^1^ Despite there being a body of biochemical and genetic mapping literature dating from the 1980s suggesting that some kind of defect in the skin barrier protein filaggrin might be involved in the pathophysiology of IV, no genetic defect had been identified to confirm or disconfirm this until some 20 years later.

The reason behind this lack of progress stems from the structure of this highly complicated protein and the very unusual gene that encodes it.^2^ The name filaggrin is a contraction of ‘filament aggregating protein’. The protein was originally identified and named by Beverly Dale in Seattle as an insoluble protein that binds keratin proteins.^3^, ^4^ Cell culture experiments showed that fluorescently‐labelled filaggrin is able to decorate keratin intermediate filaments^4^ and in addition, *in vitro* experiments showed that filaggin causes bundling and condensation of these filaments.^5^ Immunohistochemistry, immuno‐electron microscopy and further biochemical studies revealed that the precursor of filaggrin, a giant (>400 kDa) protein known as profilaggrin, is the main constituent of the keratohyalin granules that mark out the granular layer of the epidermis.^2^ The fact that these granules are visible by low‐power light microscopy in H&E‐stained skin sections is evidence that profilaggrin/filaggrin is a very highly abundant protein in the outermost layers of the epidermis. Molecular cloning of fragments of the profilaggrin mRNA and gene revealed that this encodes multiple copies of the 37 kDa filaggrin protein. Human profilaggrin consists of 10–12 near‐identical repeats of the filaggrin monomer connected by short cleavable linkers – somewhat resembling a string of sausages (Fig. 1).

**Figure 1:**
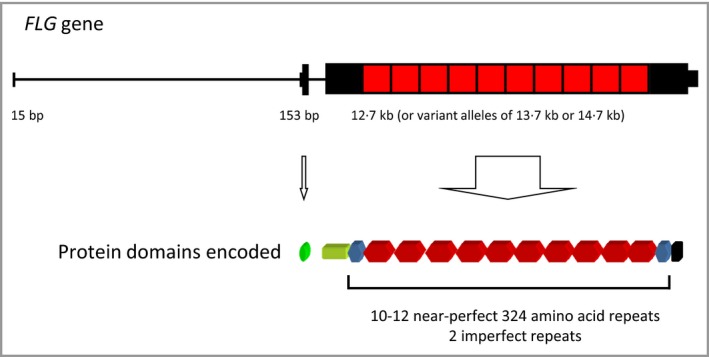
Schematic of the human profilaggrin/filaggrin gene (*FLG*) showing the very unusual intron‐exon organisation (top), colour‐coded to show of this encodes the profilaggrin protein domains (bottom). The repetitive nature of this gene and protein impeded identification of the very common *FLG* mutations for some decades.

**Figure 2:**
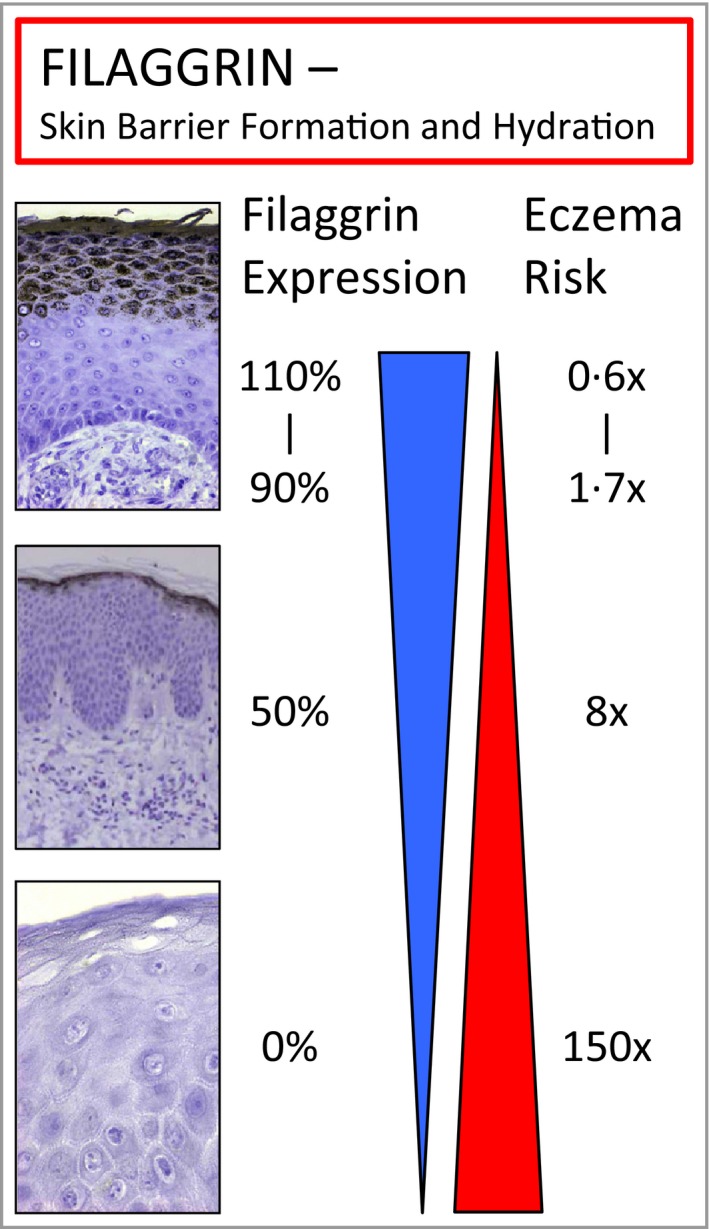
Filaggrin deficiency correlates with eczema risk. People carrying two *FLG* null mutations express zero filaggrin protein in their epidermis (bottom panel) and have a massive odds ratio of > 150, meaning that essentially all of these individuals get eczema. Heterozygous carriers of a null mutation have about 50% of the normal amount of filaggrin in their skin and have a high risk (8 × ) of developing eczema. Finally, even within the so‐called normal population, there is a ± 10% variation in the amount of filaggrin produced (due to a subtle type of sequence variation). People expressing about 90% of the normal amount of filaggrin have a small but significant eczema risk. In contrast, people expressing more than the average amount of filaggrin in their epidermis (110%), show a 40% protection from eczema (odds ratio 0·6). Thus, there is a strong correlation between the degree of genetically determined filaggrin deficency and the likelihood of developing eczema. Risks quoted here come from our original studies of filaggrin in AD 10, 14 and are higher than those reported in meta‐analyses, where in many studies only a few mutations are taken into account due to the difficulty in analysing this gene.

The proprotein is inactive within the keratohyalin granules but at the point during the epidermal differentiation programme where granular layer keratinocytes commit to forming the squames of the stratum corneum, these granules vanish as profilaggin is rapidly processed into free filaggrin monomers. This process is thought to condense the cytoskeleton and facilitate flattening of the terminally differentiating granular cells into squames. A complex cascade of phosphatases and proteases is involved in this rapid degranulation event.^2^ Within the squames, filaggrin undergoes further chemical modification and proteolytic processing to eventually broken down completely to become natural moisturizing factor (NMF) – a pool of hygroscopic amino acids and derivatives thereof that act as a natural humectant within the residual cytoplasmic space of the squames.^2^ NMF is perhaps an overly simplistic misnomer, as this material appears to have several interrelated functions including UV protection, modulating stratum corneum pH and water retention, each of which probably mediates other secondary effects.^2^ Overall, this is an important polypeptide component for the biogenesis and normal function of the skin barrier layers.

The profilaggin/filaggrin gene (*FLG*), has 3 exons and is bizarre in its sequence organisation (Fig. 1). The giant profilaggrin protein is encoded almost entirely by the enormous third exon and because this consists of several near‐identical tandem repeats of the filaggrin sequence, it was one of the last genes to be fully sequenced and assembled in the late stages of the Human Genome Project. The gene remains particularly poorly assembled and badly annotated across the many other mammalian genomes that are now available. Due to its repetitive nature, the gene is difficult to amplify by PCR and especially challenging to routinely re‐sequence for detection of pathogenic sequence variants. Even the Next‐Generation Sequencing (NGS) techniques currently in use struggle to analyse *FLG* fully and reliably.

In 2005, my laboratory, in close collaboration with Alan Irvine in Dublin and Phil Fleckman in Seattle, set about analysing the *FLG* gene for the purpose of identifying putative mutations in IV patients. One large IV family obtained from the Fleckman group had been used previously to show genetic linkage between polymorphic markers in the vicinity of the *FLG* gene and transmission of the disease through the generations of pedigree.^6^ Very confusingly, however, these data gave statistically significant linkage in that family when the disease was modelled either as a recessive condition or as a dominantly inherited trait. Normally, this should give a positive result in one model but not the other. Alan Irvine identified several Irish kindreds with IV, which crucially, his clinical team very carefully examined and phenotyped for our studies.

Using methods that we had previously developed to clone and sequence another large and unusually repetitive gene, plectin – the causative gene for epidermolysis bullosa simplex with muscular dystrophy and some other rare skin blistering conditions,^7^, ^8^ we were able to develop a strategy to sequence part of the filaggrin gene initially.^9^ In both the US kindred and in some of the Irish IV families, we identified the same nonsense mutation (R501X – arginine codon 501 mutated to a stop codon). However, in the US family, the mutation did not fully segregate with the disease and this suggested that there might be a second, as yet undetected, mutation. Using a combination of techniques and with a fair measure of good fortune, we were able to identify a second mutation in the American family, known as 2282del4 (a 4 bp deletion leading to a premature stop codon). Both the R501X and 2282del4 mutations truncate the profilaggrin protein within the first filaggrin repeat and so effectively ‘knock out’ the gene/protein more or less completely.

In human genetics, when one identifies a mutation for the first time, it is standard practice to exclude this mutation from 50 normal population controls. When we did this for the *FLG* mutations, to our great surprise, these were found at a high frequency in the co‐called normal population. Together, these mutations are carried in the heterozygous state by about 9% of the UK and Irish populations, with similar high frequencies in other populations of white Caucasian ancestry.^9^, ^10^ Very careful clinical ascertainment of the skin phenotype in several IV families in which these mutations segregate by the Irvine group revealed that the heterozygous carriers have a subtle sub‐clinical IV phenotype with dry skin that can give rise to mild scaling, particularly in cold dry climates, as well as other subtle signs of IV, such as subtle palmar hyperlinearity. Taking this into account, we showed that IV is actually a semi‐dominantly inherited condition, where heterozygous carriers have a mild IV phenotype and homozygotes or compound heterozygotes have the full‐blown classic IV phenotype.^9^ There is some degree of overlap in the symptoms depending on lifestyle and moisturiser use etc., thereby leading to confusion in the inheritance patterns seen and reported in the older literature. Thus, we had finally figured out filaggrin failure and this work was published early in 2006.^9^

Early on in the project, we realised that several of the Irish IV families also had a high incidence of atopic disease – atopic dermatitis (AD; eczema), food allergy, other allergies, asthma and hay fever. This is also reflected in the older literature. Since AD is very common and with IV also being highly prevalent, this observation could have been explained by the chance overlap of two frequent disorders within our collection of families. However, we were able to show by a number of independent and complementary genetic studies that in fact, inheritance of the filaggrin loss‐of‐function mutation confers a high risk of AD and related atopic diseases. Specifically, we able to obtain statistically significant genetic linkage between AD and *FLG* mutations within our collection of families when leaving the IV phenotype out of the equation.^11^ We were also able to show using case‐control genetic analysis that *FLG* null mutations were greatly enriched in collections of AD patients compared to ethnically‐matched control populations.^11^ In the latter studies, we obtained highly significant associations for each of the two *FLG* mutations and when these data were combined and analysed together, this gave statistical significance that was several orders of magnitude higher.

Interestingly, one of studies involved a cohort of children ascertained initially for asthma.^11^ About half of these patients also had a history of eczema. The *FLG* mutations were shown to be a major genetic risk factor for asthma *per se*, but further analysis revealed that all of the observed association was with asthma plus eczema group, where as the asthma‐only group showed no *FLG* association.^11^ This result demonstrated that there are at least two forms asthma – one driven via an eczema/skin barrier pathomechanism and the other driven by some other unknown mechanism(s). Since filaggrin is not expressed in the airway epithelia outside of the hair‐bearing outer nasal cavity (which is essentially epidermis), this led to the hypothesis that filaggrin deficiency leads to atopy via percutaneous antigen/allergen priming, which causes development of a systemic allergic immune reaction, which can then later manifest in other organ system remote from the skin, such as lung. In keeping with this, genetic associations of the *FLG* mutations were reported with hay fever and food allergy – additional phenotypic markers of the atopic diathesis.^1^ To prove this hypothesis experimentally, we turned to animal models, where we identified the causative mutation in a natural filaggrin mutant mouse known as *flaky tail*.^12^ Using these animals, we were able to show that filaggrin deficiency allows allergens cross the epidermis and trigger Th‐2 allergic immunity. Thus, the skin barrier hypothesis of eczema was established beyond all doubt and has become a widely accepted dogma in dermatology.

Importantly, our findings were quickly and widely replicated across many studies of Caucasian AD patients and our initial publication, in March 2006, is currently the most cited paper in dermatology.^11^ We went on to develop techniques to re‐sequence the entire filaggrin gene and used these methods to identify common and rare mutations in many different human populations.^10^ These mutations were causative for IV in these other ethnic groups^13^ and also strongly associated with AD across many populations, establishing filaggrin as the major genetic risk factor for AD and atopy. More recently, we showed that within the so‐called ‘normal’ population, a more subtle size variation in the *FLG* gene is also involved in eczema risk, entirely independent of the loss‐of‐function mutations.^14^

The impact of the filaggrin story was far‐reaching. The prevailing hypothesis in the AD field previously was that atopy is due to an ‘overactive’ Th‐2 immune system. While Th‐2 immunity is clearly the major mediator in the disease process, our work has shown that an important, if not essential, primary event is skin barrier deficiency. Although inherited filaggrin deficiency represents one route to a defective barrier, there are clearly others still to be worked out and this remains a fertile field within dermatology research. While the role of the barrier and the Th‐2 immune system are beyond question, a big unanswered question concerns the identity of the environmental trigger factors that drive the disease. In other words, exactly what is it out there in the environment that enters the body via a ‘leaky’ skin barrier and triggers the Th‐2 response? This may be the key to understanding another conundrum in the atopy field – why has eczema and allergy steadily increased in prevalence since the Second World War? Has this environmental trigger factor or factors increased over recent decades? Even more importantly – how can we reverse it?

A current focus of our research is to look for ways to improve skin barrier function pharmacologically and to this end, we are applying the state‐of‐the‐art drug discovery infrastructure that has become accessible to academia in recent years to the problem, with the longer term aim of translating the filaggrin discovery into direct patient benefit.
